# Virological Investigation of Avian Influenza Virus on Postglacial Species of Phasianidae and Tetraonidae in the Italian Alps

**DOI:** 10.1155/2013/601732

**Published:** 2013-09-17

**Authors:** Mauro Delogu, Giulia Ghetti, Alessandro Gugiatti, Claudia Cotti, Isabella Piredda, Matteo Frasnelli, Maria A. De Marco

**Affiliations:** ^1^Department of Veterinary Medical Sciences, University of Bologna, Via Tolara di Sopra 50, 40064 Ozzano Emilia, BO, Italy; ^2^Stelvio National Park, Via De Simoni 42, 23032 Bormio, SO, Italy; ^3^Istituto Zooprofilattico Sperimentale della Lombardia e dell'Emilia-Romagna, Via del Limite 2, 48022 Lugo, RA, Italy; ^4^Institute for Environmental Protection and Research (ISPRA), Via Ca' Fornacetta 9, 40064 Ozzano Emilia, BO, Italy

## Abstract

Land-based birds, belonging to Galliformes order are considered to be potential intermediaries in the emergence of new strains of influenza A viruses (AIVs), but the viral circulation in these birds remains largely unknown. To gain insights into the circulation of AIV in the wild Galliformes populations in Italian Alps, we conducted a virological survey on rock partridge (*Alectoris graeca saxatilis*) belonging to Phasianidae family and on tetraonids including rock ptarmigan (*Lagopus mutus helveticus*) and black grouse (*Tetrao tetrix tetrix*). In 2003 and 2004, during the hunting seasons, 79 wild *Galliformes*, categorised into age and sex classes, were hunted in the Sondrio Province (Central Alps). Cloacal swabs were collected from 11 rock partridges and from 68 tetraonids including 23 alpine rock ptarmigans and 45 black grouses. We tested cloacal swabs by a high sensitive reverse transcription- (RT-) PCR detecting the matrix gene of AIV. No AIV was detected in the investigated samples, thus, suggesting the lack of AIV circulation in these relict populations in the study period. In terms of threatened species conservation, during wildlife management activities, it is very important to exclude the introduction of AIV-carrier birds in shared territories, a fact representing a health risk for these populations.

## 1. Introduction

Global surface temperatures have increased about 0.74°C since the late nineteenth century, and the linear trend for the past fifty years of 0.13°C per decade is nearly twice that for the past hundred years [1]. As a consequence, snow extent and sea ice are projected to decrease further in the northern hemisphere, and glaciers and ice caps are expected to continue to retreat, resulting in dramatic impacts on polar and alpine environments. In these habitats, many plants and animals have already responded to this change by advancing their annual cycles [2], by modifying their distribution and the composition of communities [3]. In addition, during the last decades, human pressure on alpine wildlife habitats has significantly increased [4]. Areas above the timberline are most attractive for tourism, and disturbance by human leisure activities is considered one of the most serious threats to alpine species [5]. Moreover, the progressive abandonment of traditional farming practices as well as afforestations of high-altitude areas results in an upward advance of timberline posing serious threats to alpine and subalpine species adapted to cold habitats [6]. 

Among them, postglacial relict species belonging to Galliformes order such as rock partridge (*Alectoris graeca saxatilis*), a member of the Phasianidae family, black grouse (*Tetrao tetrix tetrix*), and alpine rock ptarmigan (*Lagopus mutus helveticus*) have recently suffered a serious numerical decline. Nowadays, in the whole Italian Alps, rock partridge population consists of 10,000–20,000 bird pairs, and those of black grouse and alpine rock ptarmigan consist of 26,000–32,000 and 5,000–8,000 individuals, respectively [7–9]. The conservation status of these species has changed in few years and, at the present time, they are listed in Annex I of the Directive 2009/147/EC of the European Parliament which aims to preserve species and their habitats. Rock partridge and alpine rock ptarmigan are also listed in the National Red Data Book of several European countries. 

In the Italian Alps, the rock partridge inhabits mountain ranges situated at elevations between 200 and 2,600 m above sea level (a.s.l.); black grouse lives at altitudes between 800 and 2,300 m a.s.l. and alpine rock ptarmigan at 1,550–3,500 m a.s.l. These wide altitude ranges are given by seasonal vertical migrations made for feeding, reproduction, and withstanding adverse climate conditions. In the nonreproductive period, during autumn and winter, alpine Phasianidae and Tetraonidae species are gregarious and congregate in monosexual groups (e.g., black grouse) or in flocks of males and females (e.g., alpine rock ptarmigan and rock partridge). 

Taking into account the great conservation concern of these alpine species and the growing interest to assess the role of infectious diseases in global species loss [10, 11], the objective of this study was to determine whether avian influenza viruses (AIV) were present in land-based birds. This group, belonging to Galliformes order, includes the species under study, chickens, quails, pheasants, and other minor poultry species considered to be potential intermediaries in the emergence of new strains of AIVs acting as potential disseminators of avian-mammalian reassortant viruses [12, 13]. Although the mechanisms of circulation of AIVs in the domestic Galliformes are well known, little and contrasting information is available about AIV ecology in free-living Galliformes populations. 

In order to estimate whether threatened alpine species such as rock partridge, black grouse, and alpine rock ptarmigan may be involved in AIV ecology, we studied these birds during the postreproductive period when (1) these species show a gregarious behaviour with an increase in interaction and AIV transmission between different flocks and (2) the peak in AIV prevalence related to the large number of young immunologically naïve birds is expected. 

## 2. Material and Methods

### 2.1. Sample Collections

**Figure 1:**
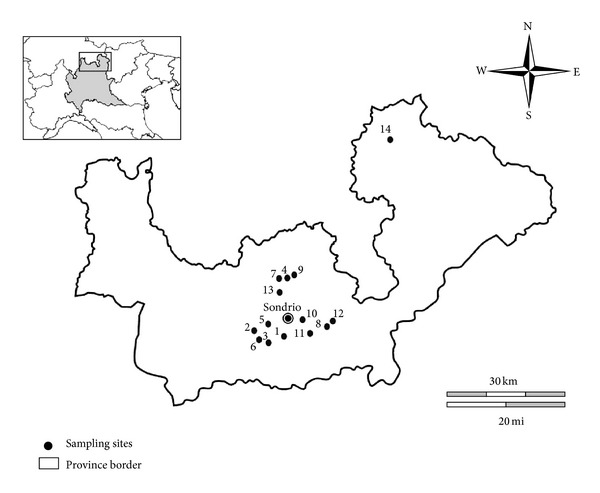
Locations of cloacal swabs sampling sites in the Sondrio Province (SO) (Central Alps, Northern Italy) collected in two post-reproductive periods (October-November) of the years 2003 and 2004. 1: Albosaggia^1,2^ (46°08′N, 9°51′E); 2: Berbenno di Valtellina^2^ (46°10′N, 9°44′E); 3: Caiolo^1^ (46°09′N, 9°48′E); 4: Caspoggio^2^ (46°15′N, 9°51E); 5: Castione Andevenno^1^ (46°10N, 9°48′E); 6: Cedrasco^1,2^ (46°9′N, 9°46′E); 7: Chiesa in Valmalenco^1,2^ (46°15′N, 9°50′E); 8: Chiuro^1,2^ (46°09N, 9°58′E); 9: Lanzada^1,2^ (46°16′N, 9°52′E); 10: Montagna in Valtellina^1^ (46°10′N, 9°54′E); 11: Piateda^1,2^ (46°09′N, 9°56′E); 12: Ponte in Valtellina^1^ (46°10′N, 9°58′E); 13: Torre Santa Maria^1,2^ (46°14′N, 9°51′E); 14: Livigno^1,2^ (46°32′N, 10°08′E). Superscripts 1 and 2 identify sites sampled in 2003 and 2004, respectively.

During the hunting season (October-November) of the years 2003 and 2004, a total of 79 free-living wild birds were hunted with the permission of the Sondrio Province (Lombardy region, Central Alps, Italy) in 14 different locations of this territory (Figure 1). The signalment (species, sex, and age), the sampling location, and the altitude were recorded by the rangers involved (Table 1).

Cloacal swabs were collected from 11 rock partridges, 45 black grouses, and 23 alpine rock ptarmigans, put in virus transport medium consisting of phosphate-buffered saline with antibiotics and glycerol (1 : 1), and stored at −20°C until laboratory testing could be conducted.

During the period of 2001–2006, in the Sondrio Province were estimated 1628 individuals of rock partridge, 2809 individuals of black grouse, and 1137 individuals of alpine rock ptarmigan [14]. Taking into account these estimation sizes, our sampling represented the 0.7%, 1.6%, and 2% of rock partridge, black grouse, and alpine rock ptarmigan population of the Sondrio Province, respectively. As a result of further populations decline, in last years, hunting has been banned or severely restricted in many areas of the Italian Alps.

### 2.2. Viral Analysis

To detect AIV we used a very sensitive and accurate one-step reverse transcription- (RT-) PCR detecting the matrix (M) gene of influenza A virus [15]. Pools of five cloacal samples were prepared and processed as previously described [16]; a positive control and two negative controls were included. 

RNA extraction was conducted by using a QIAamp Viral RNA Mini Kit (Qiagen, Hilden, Germany) according to the manufacturer's recommendations. Samples were amplified in one-step RT-PCR and amplicons were separated by gel electrophoresis through a 2% (w/v) agarose gel, stained with GelRed (Biotium Inc., Hayward, CA), and analysed by a GelDoc-It quantitative imaging system (UVP, LCC Upland, CA).

## 3. Results and Discussion 

During the study, a total of 79 cloacal swabs were collected from rock partridge, black grouse, and alpine rock ptarmigan in the Italian Alps. Samples tested using a very sensitive RT-PCR targeting the M gene gave negative results, indicating that no AIV could be detected in these Phasianidae and Tetraonidae species examined in two consecutive post-breeding seasons (Table 1). Positive and negative control samples revealed appropriate product and no product, respectively, indicating correctly performed molecular processes (correct PCR conditions and no RNA contaminations). 

Although AIV infections were described in pheasants reared in Italy (*Phasianus colchicus*) during limited outbreaks [17] or associated with severe poultry epidemics [18], the occurrence of AIV infection in free-living Galliformes species was sporadically reported in pheasants in Europe [19] and in rock partridges in Israel [20]. Several studies described that turkeys, pheasants, Japanese quails, and red-legged partridges, belonging to Phasianidae family, are more susceptible than chickens to AIV infection transmitted from free-living aquatic birds [13, 21]. Furthermore, experimental infections showed that highly pathogenic (HP) AIVs could cause specific clinical signs and mortality in the above-mentioned species [21, 22] and that pheasants are long-term shedders of low pathogenic AIV [23, 24]. 

In addition to the AIV susceptibility demonstrated in Galliformes species closely related to those examined in the present study, other ecological factors could facilitate the potential AIV circulation. In particular, the increased bird flock density related to their gregarious behaviour in the postbreeding period, the presence in these groups of juvenile birds more susceptible than adults to the infection [25], and the autumn vertical migrations which increase the opportunities of contact with other cospecific wild or domestic birds living at a much lower altitude may enhance AIV population exposure. In addition, humid weather and cool temperatures, typical of alpine habitats, may contribute to environmental survival of the virus [26, 27] allowing possible new infections for a long period of time.

In such a context, the virological absence of AIV detection in these populations could be explained taking into account that the opportunities of interaction in shared water sources with potentially infected migratory reservoir species are very low [13]. Hence, we may exclude the possible circulation and spread of the AIVs within these wild Galliformes populations during the periods when the peak in AIV prevalence is expected. However, having no such possibility to carry out serological investigation, we cannot rule out that single, accidental infections might occur in breeding season, when the species is monogamous and cocks use large home ranges, as observed in the Italian Alps [28]. 

Although a recent epidemiological theory [29] predicts that one of the most commonly cited factors for disease-induced extinction is the presence, in the same territory, of threatened population and reservoir hosts species, other theoretical mechanisms that could produce disease-induced extinction are small preepidemic population size of the endangered species and long-term survival of the infectious agent in the abiotic environment.

In addition to these specific circumstances, as observed in other threatened species [30], the combination of potential population decline causes, such as the exposure of host population to HPAIV [31] and the introduction of domestic or wild AIV-carrier birds [32], possibly related to animal release for restocking purposes [11] could represent a health risk for these endangered relict species exposing them to so far unencountered infections, with unknown consequences for the species survival. Although host species should preferably be sampled in large numbers to get estimates reflecting the true prevalence, for small populations of elusive species living in areas that are often difficult to access [33], this is not possible. To our knowledge this study, even if limited in number, represents the first research of AIV infection in alpine Galliformes species. 

## Figures and Tables

**Table 1:** Overview of free-living Galliformes species collected during the hunting season in two postreproductive periods of the years 2003 and 2004 sampled by location and altitude in the Sondrio-province (Lombardy region, Central Alps). Birds are categorised into sex and age classes. Results of RT-PCR assay specific for the detection of AIVs are also shown.

Species	Sampling years	Sampling sites^a^	Sampling altitude (m a.s.l.)	Sex classes^b^	Age classes^c^	Total	RT-PCR positive/total examined
Rock partridge (*Alectoris graeca saxatilis*)	2003	9, 10, 11	1400–2000	M: 1 F: 3 I: 2	Juv: 6 Ad: 0	11	0/11
	2004	11, 13	1600–2300	M: 3 F: 2 I: 0	Juv: 3 Ad: 2		
Black grouse (*Tetrao tetrix tetrix*)	2003	1, 3, 5, 6, 7, 8, 9, 10, 11, 12, 13, 14	1700–2200	M: 38 F: 0 I: 0	Juv: 25 Ad: 13	45	0/45
	2004	1, 2, 4, 6, 9, 14	1800–2000	M: 7 F: 0 I: 0	Juv: 2 Ad: 5		
Alpine rock ptarmigan (*Lagopus mutus helveticus*)	2003	7, 8, 10, 14	2600–3000	M: 6 F: 5 I: 0	Juv: 4 Ad: 7	23	0/23
	2004	7, 8	2500–2900	M: 6 F: 6 I: 0	Juv: 6 Ad: 6		

^a^Site numbers as given in Figure 1. ^b^M: male; F: female; I: undetermined. ^c^Juv: juvenile; Ad: adult.
